# Teachers' digital competences: a scale construction and validation study

**DOI:** 10.3389/fpsyg.2024.1356573

**Published:** 2024-08-16

**Authors:** Mehmet Kemal Aydin, Turgut Yildirim, Metin Kus

**Affiliations:** ^1^Distance Education, Research and Application Centre, Hitit University, Çorum, Türkiye; ^2^Department of Physical Education Teacher Education, Faculty of Sport Sciences, Hitit University, Çorum, Türkiye

**Keywords:** digital competence, reliability, scale development, teachers, validation

## Abstract

**Introduction:**

Improving teachers' digital competences is sine qua non for effective teaching and learning in today's digital society. However, there is a limited number of comprehensive and reliable scales to measure teachers' digital competences. Regarding this, the present study aimed to develop and validate a comprehensive scale to assess teachers' digital competences.

**Methods:**

Building on previous studies, a draft scale developed and piloted with a sample of teachers from all educational levels. The procedures of Exploratory Factor Analysis (EFA) were followed to refine the scale, resulting in a five-point Likert scale with 36 items loaded onto four factors. The final scale was called as Teachers' Digital Competences Scale (TDC-S). Confirmatory factor analysis (CFA) was employed to validate the four-factor structure. Reliability analysis was performed using Cronbach's alpha (α), McDonald's omega (ω), and Composite Reliability (CR), indicating high psychometric properties. Convergent and discriminant validity analyses were also performed to assess the validity of the latent structures in TDC-S.

**Results and discussion:**

The findings suggest that the TDC-S is a valid and reliable instrument for assessing teachers' digital competences at all grade levels from primary to high schools. It can be used to inform teacher training and development programs, and to identify teacher candidates who need additional assistance regarding improving their digital competences.

## 1 Introduction

Digital technologies have had a profound impact on society and learning culture over the past two decades. This has necessitated the adaptation of schools and society to this digital transformation, which was further underscored by the COVID-19 pandemic. In response, many countries have prioritized the digitalization of society and education, developing frameworks and tools to improve digital competency (Fernández-Miravete and Prendes-Espinosa, 2022). In today's digital society, it is essential for every citizen to acquire digital skills as a core competence for personal development, socialization, employment, and lifelong learning (Council of the European Union, 2018; Rodríguez-García et al., 2019). Digital competence and digitalization are expected to play crucial roles in shaping the future economic and social landscape of Europe. Thus, recognizing its growing importance, the European Council has highlighted digital competence as a fundamental skill necessary for personal fulfillment, promoting a healthy lifestyle, ensuring employability, fostering active citizenship, and facilitating social inclusion. This strategic focus on digital competence is in line with the evolving educational needs and trends, emphasizing the critical role that digital literacy plays in contemporary society (European Commission, 2023). The European Commission's proactive approach to integrating digital competence into essential competencies for individuals demonstrates a forward-looking strategy aimed at equipping citizens with the skills needed to thrive in an increasingly digitalized world. By prioritizing digital competence as a key competence, the European Council not only acknowledges the transformative potential of digital skills but also shows a commitment to preparing individuals for the challenges of the digital age (Castaño Muñoz et al., 2023; Council of the European Union, 2018). However, digital transformation presents some major challenges for education systems, including digital literacy and the quality of education. Improving teachers' and students' digital competences is essential to achieve digital transformation effectively in educational settings (Martín-Párraga et al., 2023). In this context, educators at all levels of education have a great responsibility to raise qualified and creative individuals. Thus, teachers need to update their competency profiles and teaching strategies to align with the requirements of the digital society (Caena and Redecker, 2019; Gómez-García et al., 2022).

Although the impact of technology in the teaching-learning process has become more evident in the last few years, there are many challenges education systems should overcome for an effective integration of digital technologies into this process. Several influencing factors and challenges can be listed as such the use of virtual learning environments, emerging technologies, digital platforms, and social networks and so forth (Garzón Artacho et al., 2020). To achieve the goal of sustainable and quality education, teachers need to be competent enough to meet the educational demands placed on them (Mafratoglu et al., 2023). This has led to the development of not only teachers' digital competences (TDC) but also students' digital competences and the utilization of digital technology to improve education (Ghomi and Redecker, 2019). Teachers who were exposed to technology in the middle of their professional lives, especially during the COVID-19 pandemic period, had to use various educational tools such as e-textbooks, internet technologies, exam programs and educational resources (Yelubay et al., 2020). Caught unprepared, teachers have faced a lack of understanding of digital technology, which has caused a huge gap in the education system (Ochoa Fenández, 2020). Therefore, the challenge of preparing teachers to ensure the effective and efficient use of digital technology in schools remains unclear. The evolving nature of technology requires educators to continuously develop their skills and digital competences to keep pace with the rapidly changing educational realm (Falloon, 2020).

The integration of ICTs in the educational context has prompted the development of policies to address one of the pedagogical challenges, namely improving teachers' digital literacy (Garzón Artacho et al., 2020). In this regard, particularly the emergence of “The European Framework for the Digital Competence of Educators” (DigCompEdu) in 2017 has catalyzed the formulation and implementation of policies by various nations to assess and enhance TDC (Christine, 2017). The updated version of DigCompEdu framework proposes a structured six-point progression model for appraising educator competence proficiency. The framework outlines six distinct levels of TDC from A1 (Awareness) to C2 (Innovation). Concurrently with the introduction of DigCompEdu, there has been a salient surge in research endeavors focusing on the examination of TDC (Aydin and Yildirim, 2022; Madsen et al., 2023). To facilitate the acquisition of digital competency among educators, several competence frameworks have been proposed. All these frameworks aim to explore the way in which technologies should be integrated and used in teaching in order to identify educational needs and propose personalized educational programs (Flores-Lueg and Roig Vila, 2016).

TDC and underpinning frameworks are essential for effective teaching and learning in the 21st century. These frameworks provide a clear and comprehensive roadmap for teacher development, supporting the development of school-wide digital learning initiatives, and enabling the pathways to the assessment of the impact of teacher professional development programs. In line with this, several organizations have attempted to identify and develop TDC. These efforts include European Digital Competence Framework for Educators, DigCompEdu; ISTE standards for Educators; UNESCO ICT Competence Framework for Teachers; Spanish Common Framework of Teacher's Digital Competence; British Digital Teaching Professional Framework; Colombian ICT Competences for teachers' professional development; Chilean ICT Competences and Standards for the teaching profession. However, Cabero-Almenara et al. (2020b) evaluated seven most commonly used TDC frameworks and concluded that the DigCompEdu is the most appropriate model for assessing TDC. The structured breakdown of DigCompEdu offers a robust framework for assessing educators' competencies, enabling a detailed examination of their digital skills and capabilities within the educational domain. This hierarchical model categorizes educators into discrete stages, facilitating a systematic approach to evaluating and enhancing educator competence (Cabero-Almenara et al., 2020b). By delineating competence levels in this manner, the model supports the continuous professional development (CPD) of teachers, aligning with the evolving demands of the educational landscape (Santo et al., 2022). In line with this, many researchers have employed DigCompEdu as a framework to develop instruments in order to assess TDC (Alarcón et al., 2020; Cabero-Almenara and Palacios-Rodríguez, 2020; Ghomi and Redecker, 2019). Building upon the DigCompEdu framework, SELFIEforTEACHERS has been develop more recently as a self-reflection tool designed to support primary and secondary teachers in developing their digital capabilities. Through a self-reflective process, teachers are able to self-assess their digital competence, pinpointing both strengths and areas for improvement. The feedback provided by this tool empowers teachers to actively engage in their professional learning journey, fostering the integration of digital technologies within their professional context (European Commission, 2023). Additionally, this tool empowers teachers to take charge of their professional growth and development. By fostering a culture of self-directed learning, SELFIEforTEACHERS aligns with contemporary educational paradigms that emphasize the importance of personalized and continuous professional development for educators.

In a similar vein, in the present study, DigCompEdu served as a theoretical framework to inform the creation of item pool and the development of draft scale. In addition to the value of these frameworks, the scale development studies pertinent to assessment of TDC are salient since these tools can provide invaluable information regarding teachers' knowledge, skills, and attitudes regarding digital technologies (Rodríguez-García et al., 2019). This information can be used to identify areas where teachers need additional support and to provide them feedback on self-direct their own continuous professional learning. Furthermore, measuring teachers' digital competencies is essential for ensuring effective integration of digital resources in educational settings, enhancing teaching practices, and preparing educators to meet the demands of digital learning environments. There are a variety of different TDC measurement tools available (Alarcón et al., 2020; Al Khateeb, 2017; Cabero-Almenara et al., 2020a; Ghomi and Redecker, 2019; Gümüş and Kukul, 2023; Kuzminska et al., 2019; Tondeur et al., 2017; Tzafilkou et al., 2022). All these efforts delineate the growing importance of teachers' digital competency, which is among the most important competences that teachers should master in today's digital society (Aydin and Yildirim, 2022; Cabero-Almenara and Martínez, 2019; Cabero-Almenara and Palacios-Rodríguez, 2020).

Given this context, although a number of assessment tools have been devised to assess TDC, a comprehensive synthesis and critical evaluation of these tools are lacking. As a response to this need, in a recent review study, Nguyen and Habók (2024) systematically reviewed the 33 TDC scales sourced from peer-reviewed journals indexed in prominent databases, such as the Education Resources Information Center (ERIC), Web of Science (WoS), and Scopus. The time frame for the study was from 2011 to 2022 and the search terms included “ICT competency” or “ICT literacy” or “digital literacy” or “information literacy” or “computer literacy” or “technology literacy” and “assessment”. The study aimed to discern prevalent evaluation aspects, types of assessment tools employed, and the reported reliability and validity of these tools in addition to the frameworks or models underpinning the design of these assessment tools. The analysis revealed a predominant research focus on digital competence in teachers' utilization of educational technology, integration of ICT in teaching and learning, professional development, and support mechanisms for learners through digital competence. Results suggested that future research endeavors should aim at advancing the exploration of TDC. The study also purported that there's no perfect assessment tool for TDC, making it tough to gauge teachers' or students' skills accurately. To create effective assessment tools, researchers should consider factors like where the study takes place, who's participating, and the available resources. Additionally, since technology keeps changing, there is a need to update these assessment tools regularly to keep up with new advancements. As a result, despite the availability of several tools to assess TDC, there is a need for further tools that are more comprehensive and practical, and that can be used to measure the digital competence of teachers and empirically tested in different contexts (Nguyen and Habók, 2024). The selection of appropriate measurement tool for a particular context will depend on the specific needs of the researchers, teachers and the school or district. This highlights the need for a practical and comprehensive scale to assess TDC. Moreover, existing scales often focus on subject-specific digital competences (Al Khateeb, 2017) and primarily target university faculty members (Cabero-Almenara et al., 2020a), undergraduate and graduate students (Tzafilkou et al., 2022), and teacher candidates (Tondeur et al., 2017). Unlike the previous studies, the present study addresses these limitations by incorporating a sample group of teachers from diverse branches and levels, ranging from primary to upper-secondary grade levels. This empirical originality highlights comprehensiveness, practical applicability, as well as the rationale of the current study. Consequently, by developing and validating a comprehensive scale that measures TDC, the present study holds significant contributions to both theoretical and practical aspects of the educational technology research realm. On the practical side, by using TDC-S, teachers can self-assess their digital competence, identifying both strengths and areas for improvement as well as providing feedback for self-directing their continuous professional learning journey, in addition to fostering the integration of digital technologies within their professional context.

In the present study, our aim is to develop and validate a contemporary measurement tool, building upon previous reports and studies in the field. To align with this overarching goal, we have outlined specific research objectives: (1) To examine the validity and reliability of the Teachers' Digital Competence Scale. (2) To evaluate and confirm both the exploratory and confirmatory factor structures of the Teachers' Digital Competence Scale.

## 2 Materials and methods

### 2.1 Development process of TDC scale

This study employed a quantitative research method, specifically a descriptive survey design, to develop the Teachers' Digital Competence Scale (TDC-S). The development of the scale followed an 8-step framework proposed by DeVellis (2017). This framework ensures the reliability, validity, and comprehensiveness of the resulting measurement tool. The stages of development include identifying conceptual bases, generating an item pool, determining the format for measurement, obtaining expert review for the item pool, considering the inclusion of validation items, administering items to a development sample, evaluating the items, and optimizing the scale length (DeVellis, 2017).

### 2.2 Participants

The study was conducted in the 2022–2023 academic year with a sample of teachers from different educational levels and branches in Çorum, Türkiye. Convenience sampling was used to select the participants. Data were collected through online and face-to-face survey techniques, ensuring voluntary participation, and protecting the privacy and confidentiality of the participants. An informed consent was obtained from the participants, and they were informed about the purpose of the study and the conditions of participation. In addition, a letter of approval was retrieved from the Ethics Committee Review Board of Hitit University (2021-88/10.01.2022). As a result, the study was carried out in accordance with the guidelines set forth in the Declaration of Helsinki. All participants took part in the surveys on their own free will, and no incentives were given to encourage participation.

The initial scale form consisted of 46 items developed building on previous studies in the literature (Alarcón et al., 2020; Al Khateeb, 2017; Cabero-Almenara et al., 2020a; Ghomi and Redecker, 2019; Gümüş and Kukul, 2023; Kuzminska et al., 2019; Tondeur et al., 2017; Tzafilkou et al., 2022), expert opinions and feedback. A total of 271 teachers participated in the study. Validity and reliability analyses were conducted. The demographic characteristics of the participants are presented in Table 1.

**Table 1:** Demographic characteristics of participants.

***Variables***	***Groups***	***f***	***%***
Gender	Female	144	53.1
	Male	127	46.9
Age	20–29	26	9.6
	30–39	88	32.5
	40–49	128	47.2
	50 and above	29	10.7
Teaching experience	1–10	63	23.2
	11–20	114	42.1
	21–30	84	31.0
	31 and above	10	3.7
Subject disciplines	Class teachers	17	6.3
	Foreign languages	24	8.9
	Guidance and counseling	14	5.2
	ICT	11	4.1
	Language and literacy	24	8.9
	Maths	36	13.3
	Others	22	8.2
	PE	59	21.8
	Religion	12	4.4
	Science	21	7.7
	Social sciences	16	5.9
	Special needs education	15	5.5
School type	Primary	29	12.2
	Lower-secondary	135	49.8
	Upper-secondary	103	38.0
Perceived ICT skills	Low	32	10.7
	Moderate	115	42.4
	Competent	127	46.9
ICT use frequency	Rarely	26	9.5
	Occasionally	75	27.7
	Frequently	126	46.5
	Always	44	16.2

Upon analysis of the demographic data presented in Table 1, it is evident that the study group consisted of 271 teachers, with 53% female and 47% male participants. The age distribution of the participants was as follows: 10% were aged 20–29, 32% were aged 30–39, 47% were aged 40–49, and 11% were aged 50 and above, with a mean age of 40.61 (SD=7.57). In terms of teaching experience, 23% of the participants had 1–10 years of experience, 42% had 11–20 years, 31% had 21–30 years, and 4% had 31 years teaching experience and above, with a mean seniority of 16.70 (SD=7.96). In line with the research aim, the participants represented a wide range of subject disciplines, including class teachers and special needs education teachers. They were employed in primary schools (12%), lower-secondary schools (50%), and upper-secondary schools (38%). Regarding the perceived ICT skills of the participant teachers, the findings indicated that 11% reported insufficient competence in ICT use, while 42% reported a moderate level of competency. The majority of participants (47%) reported a high level of competency in ICT use. The frequency of ICT use in their classes varied, with 10% reporting rare use, 28% reporting occasional use, 46% reporting frequent use, and 16% reporting constant use during classes.

### 2.3 Analysis of data

The validity and reliability of the Teachers' Digital Competence Scale were examined. Before conducting the analyses, the normality assumptions of the data were evaluated by examining the skewness and kurtosis values and it was seen that the data were normally distributed. Then, Kaiser-Meyer-Olkin (KMO) coefficient and Bartlett's test were performed to evaluate the suitability of the data for factor analysis. Kaiser (1974) eloquently stated that Kaiser-Meyer-Olkin (KMO) values in the 0.90s are superb, in the 0.80s are commendable, in the 0.70s are adequate, in the 0.60s are mediocre, in the 0.50s are unsatisfactory, and below 0.50 are unacceptable (Kaiser, 1974). After checking the appropriateness of the data set for factor analysis, an Exploratory Factor Analysis (EFA) and a Confirmatory Factor Analysis (CFA) were performed to evaluate the construct validity of the scale. Internal consistency coefficients [Cronbach's alpha (α), McDonald's omega (ω), and Composite Reliability (CR)] were estimated to assess the reliability of the scale. In addition to internal consistency coefficients, convergent and discrimination validity tests such as CR, Average Variance Extracted (AVE), and Heterotrait-Monotrait (HTMT) correlations ratio were performed to assess the psychometric quality of the scale.

The primary objective of this study was to explore the latent structure of the 46 items. To achieve this goal, a common factor model, specifically Exploratory Factor Analysis (EFA), was employed as the appropriate statistical technique (Watkins, 2021). EFA is a common method used in research to identify latent factors that explain the observed variance in a set of variables. In this study, squared multiple correlations (SMCs) were used to estimate the initial communality values (Tabachnick and Fidell, 2019). SMC is a measure that indicates the proportion of variance in an observed variable that can be accounted for by the common factors. It provides an initial estimate of the shared variance among the variables before the factor analysis is conducted (Watkins, 2021). By employing EFA and using SMC for initial communality estimates, this study aimed to uncover the latent structure and understand the underlying relationships among the measured variables. While EFA was used to determine the underlying factor structure of the scale, CFA was used to confirm the factor structure proposed in EFA. The methodological choices in this study were designed to ensure a rigorous and comprehensive analysis of the data, resulting in a reliable and valid measurement tool for assessing TDC.

## 3 Results

In this section, the findings of the validity and reliability studies conducted as part of the development of the instrument were presented. Exploratory factor analysis (EFA) and Confirmatory factor analysis (CFA) were conducted to demonstrate construct validity. The internal consistency coefficients and convergent and discrimination validity tests were performed to assess the psychometric quality of the scale.

### 3.1 Exploratory factor analysis (EFA)

Exploratory factor analysis (EFA) is a multivariate statistical technique that has become a cornerstone in the development and validation of measurement tools. However, the application of EFA requires researchers to make several methodological decisions, each of which can have a significant impact on the results. These decisions include the choice of factor extraction method, rotation method, and factor loading threshold, among others. There is no one-size-fits-all approach to EFA, and the best choices for each decision will vary depending on the specific research context. However, researchers should carefully consider the available options and make their decisions based on sound theoretical and empirical grounds (Watkins, 2021).

An EFA was performed to ensure the construct validity of the Teachers' Digital Competence Scale. Before performing EFA, the dataset used in this study was checked if it is appropriate for factor analysis based on several indicators. Firstly, the sample size of 271 participants met the recommended criteria for factor analysis. A sample size of this magnitude is considered suitable for conducting factor analysis (Bryant and Yarnold, 1995; Tabachnick and Fidell, 2001). Furthermore, the correlation matrix of the data was inspected to assess the inter-correlations among the scale items. It was found that the majority of inter-correlations exceeded the threshold of <0.3 (Tabachnick and Fidell, 2001). This indicates that there is a sufficient level of association between the items, which is a prerequisite for factor analysis. In addition, Bartlett's test of sphericity was conducted to evaluate the interdependence among the items. The test yielded a significant result (*X*^2^ = 8,916,504, *df* = 630, *p* < 0.05), suggesting that the items in the dataset are not independent and are suitable for factor analysis (Bartlett, 1954). Lastly, the Kaiser-Meyer-Olkin (KMO) sampling statistic was calculated to assess the adequacy of the dataset for factor analysis. The KMO value of 0.965, which exceeds the recommended threshold of 0.70 (Kaiser, 1974), indicates that the dataset is highly suitable for factor analysis. In summary, all these indicators provide confidence in the suitability of the dataset for conducting factor analysis.

After confirming the EFA assumptions, a principal axis factor analysis (PAF) with oblique rotation was conducted. Oblique rotation was chosen to allow for the possibility of correlated factors, which is appropriate when the factors are expected to be related or correlated. PAF is a method of extracting factors from a correlation matrix, and oblique rotation is a method of rotating the factors in such a way that they are allowed to correlate with each other. This is in contrast to orthogonal rotation, in which the factors are forced to be uncorrelated (Watkins, 2021). It is noteworthy that the factor structure analysis was conducted without imposing any restrictions, allowing the scale items to load on any number of factors. This approach provides flexibility in capturing the underlying structure of the data and allows for a more accurate representation of the TDC. By employing an oblique rotation and not imposing any restrictions on the factor structure, the analysis aimed to capture the complex relationships and potential interdependencies among the scale items. This approach is supported by the literature, which emphasizes the importance of considering the design and analytical decisions in factor analysis and the consequences they have on the obtained results (Fabrigar et al., 1999; Li et al., 2015).

Prior to conducting exploratory factor analysis (EFA), researchers should establish a threshold for factor loadings to be considered meaningful (Worthington and Whittaker, 2006). Pattern coefficients for oblique rotation loadings that meet this threshold are defined as salient. It is common practice to arbitrarily consider factor loadings of 0.30, 0.32, or 0.40 as salient (Hair et al., 1998), meaning that variables with around 9%, 10%, or 16% (factor loading squared) of their variance is explained by the factor. Some researchers consider 0.30 or 0.32 to be salient for EFA and 0.40 to be salient for PCA (Watkins, 2021). These thresholds reflect practical significance but do not guarantee statistical significance. A loading of 0.32 may account for 10% of a variable's variance, yet it may not be statistically significantly different from zero (Zhang and Preacher, 2015). In this regard, Norman and Streiner (2014), suggested an approximation based on Pearson correlation coefficients to calculate the statistical significance (*p* = 0.01) of factor loadings (Norman and Streiner, 2014). For the TDC scale, statistical significance (*p* = 0.05) would equate to 0.32. Therefore, in this study, a loading of 0.32, which may account for 10% of a variable's variance, was specified as a threshold for salient loadings (Watkins, 2021). In line with this, preliminary analysis purported that 10 items should be removed from the item pool. The items 4, 19, 36, and 45 had a factor loading below cut-off value of 0.32. Additionally, the items 7, 25, 28, 30, and 34 were also removed since they are overlapping loadings which is <0.10. As a result of the preliminary analysis these 10 items were removed to refine scale items prior to EFA as suggested by Tabachnick and Fidell (2019) and Watkins (2021).

**Figure 1:**
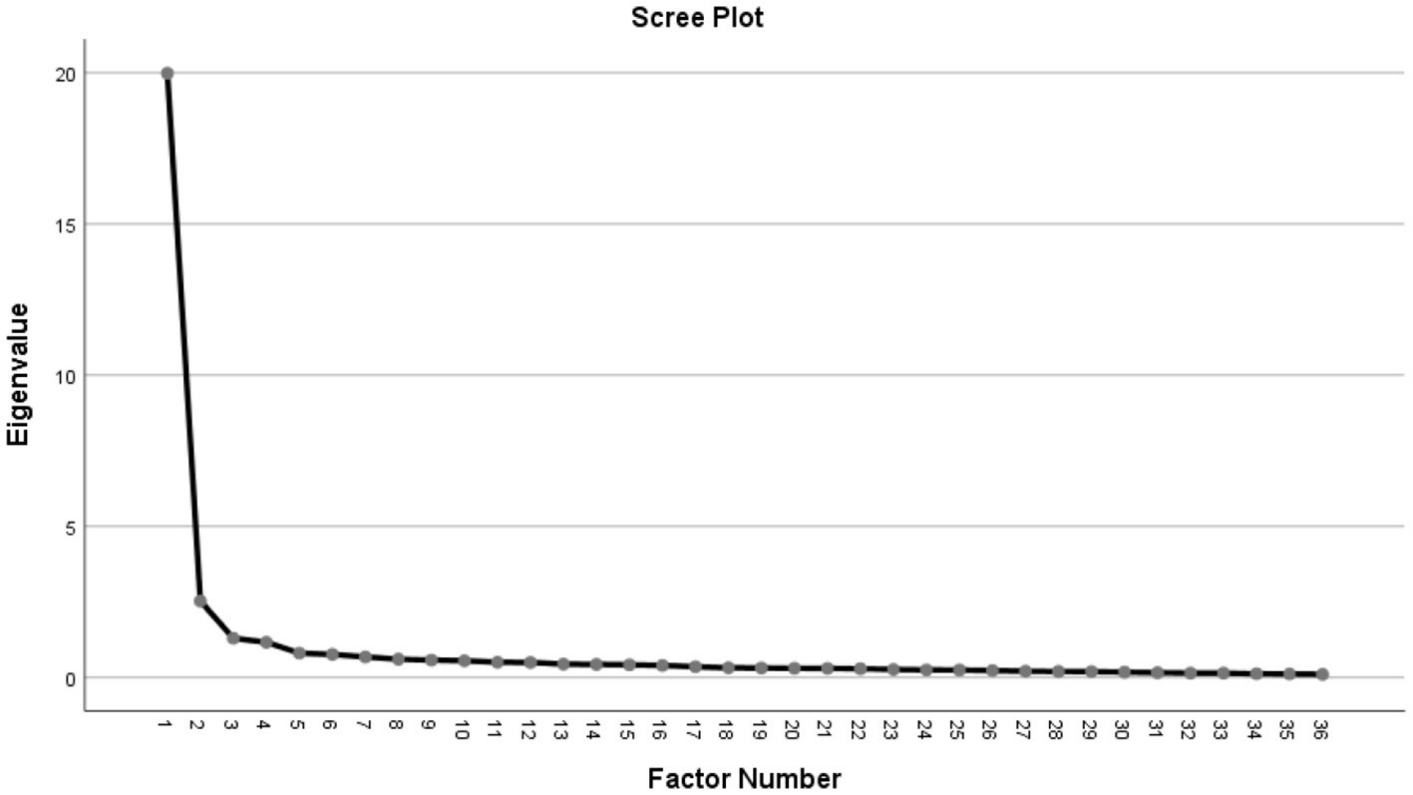
Scree plot output for retained factors based on eigenvalues.

EFA outputs are presented in Table 2, Figure 1. The factor structure of the scale was examined by analyzing the eigenvalues, salient loadings, total variance explained and scree plot. The scree plot of the eigenvalues is presented in Figure 1. The EFA results provided evidence for the construct validity of the Teachers' Digital Competence Scale because the factor structure of the scale was found to be consistent with the underlying theoretical framework, DigCompEdu. EFA is a widely accepted method for assessing the construct validity of measurement instruments and was used in this study to validate the Teachers' Digital Competence Scale.

**Table 2:** Distributions of items after rotation.

**Factor**	**Item**	**Initial eigenvalues**	**Common variance**	**Promax Rotated**			
				**Factor 1**	**Factor 2**	**Factor 3**	**Factor 4**
Factor 1	24	0.759	0.715	0.968			
	33	0.734	0.654	0.908			
	26	0.698	0.641	0.844			
	41	0.721	0.664	0.753			
	44	0.704	0.652	0.718			
	46	0.706	0.641	0.706			
	32	0.777	0.725	0.699			
	18	0.739	0.687	0.696			
	02	0.704	0.653	0.689			
	23	0.662	0.546	0.681			
	27	0.716	0.640	0.651			
	29	0.796	0.700	0.638			
	13	0.747	0.725	0.638			
	11	0.772	0.712	0.630			
	10	0.790	0.755	0.603			
	22	0.686	0.594	0.602			
	12	0.586	0.524	0.592			
	01	0.655	0.546	0.545			
Factor 2	39	0.780	0.741		0.880		
	38	0.742	0.729		0.869		
	40	0.820	0.797		0.813		
	42	0.540	0.497		0.731		
	37	0.673	0.634		0.721		
	43	0.732	0.712		0.590		
	31	0.735	0.710		0.554		
	35	0.703	0.652		0.548		
	08	0.641	0.556		0.477		
	20	0.697	0.650		0.379		
Factor 3	05	0.739	0.727			0.786	
	06	0.770	0.760			0.691	
	09	0.755	0.710			0.590	
	03	0.497	0.394			0.415	
Factor 4	15	0.747	0.792				0.885
	16	0.692	0.707				0.777
	14	0.706	0.694				0.723
	17	0.582	0.482				0.351

Total variance explained: %65.757.

Explained variance for Factor-1: %17,810.

Explained variance for Factor-2: %14,428.

Explained variance for Factor-3: %12,864.

Explained variance for Factor-4: %12,155.

The scree plot in Figure 1 suggested a four-dimension structure with eigenvalues above 1. The initial eigenvalues of the first, second, third, and fourth factors are 19.981, 2.527, 1.295, and 1.161, respectively. The number of factors with eigenvalues above 1 indicates the number of components.

When the total variance explained is examined, it was seen that the first factor explained 24.262% of the total variance, while the second, third, and fourth factors explained 17.810%, 14.428%, 12.864%, and 12.155% of the factor variance, respectively. The total variance explained by the four factors together is 65.757%. These values are within the variance ratios (2/3) that multifactor scales should account for social science research as suggested by Hair et al. (2019).

Promax output was examined to determine which items fell under each of the four factors. The EFA results provided evidence for the construct validity of the TDC Scale, as the factor structure of the scale was found to be consistent and mostly overlapping with the dimensions of DigCompEdu framework.

When the distribution of the Promax rotated items in the Teachers' Digital Competence Scale was examined, the first factor consisted of 18 items and labeled as “Teachers' Professional Digital Competence” (TPDC), the second factor consisted of 10 items and labeled as “Teachers' Use of Instructional and Communication Tools” (TUICT), the third factor consisted of 4 items and labeled as “Teachers' Use of Hardware Tools” (TUHT), and finally the fourth factor consisted of 4 items and labeled as “Teachers' Digital Content Development” (TDCD). The factor loadings of the items in the first factor ranged between 0.968 and 0.545. Factor loadings of the items in the second, third, and fourth factors ranged between 0.880 and 0.379, 0.786, and 0.415, and 0.885 and 0.351, respectively. The results of the analysis showed that there were no overlapping items in the scale and no item had a factor loading below 0.32. Based on the eigenvalues, explained variance and the scree plot, it was concluded that the TDC-S may have a four-factor latent structure. However, it is important that this structure is statistically demonstrated and theoretically justified for the convenience of researchers. Therefore, the four-factor structure proposed by EFA was tested using CFA.

### 3.2 Confirmatory factor analysis

**Figure 2:**
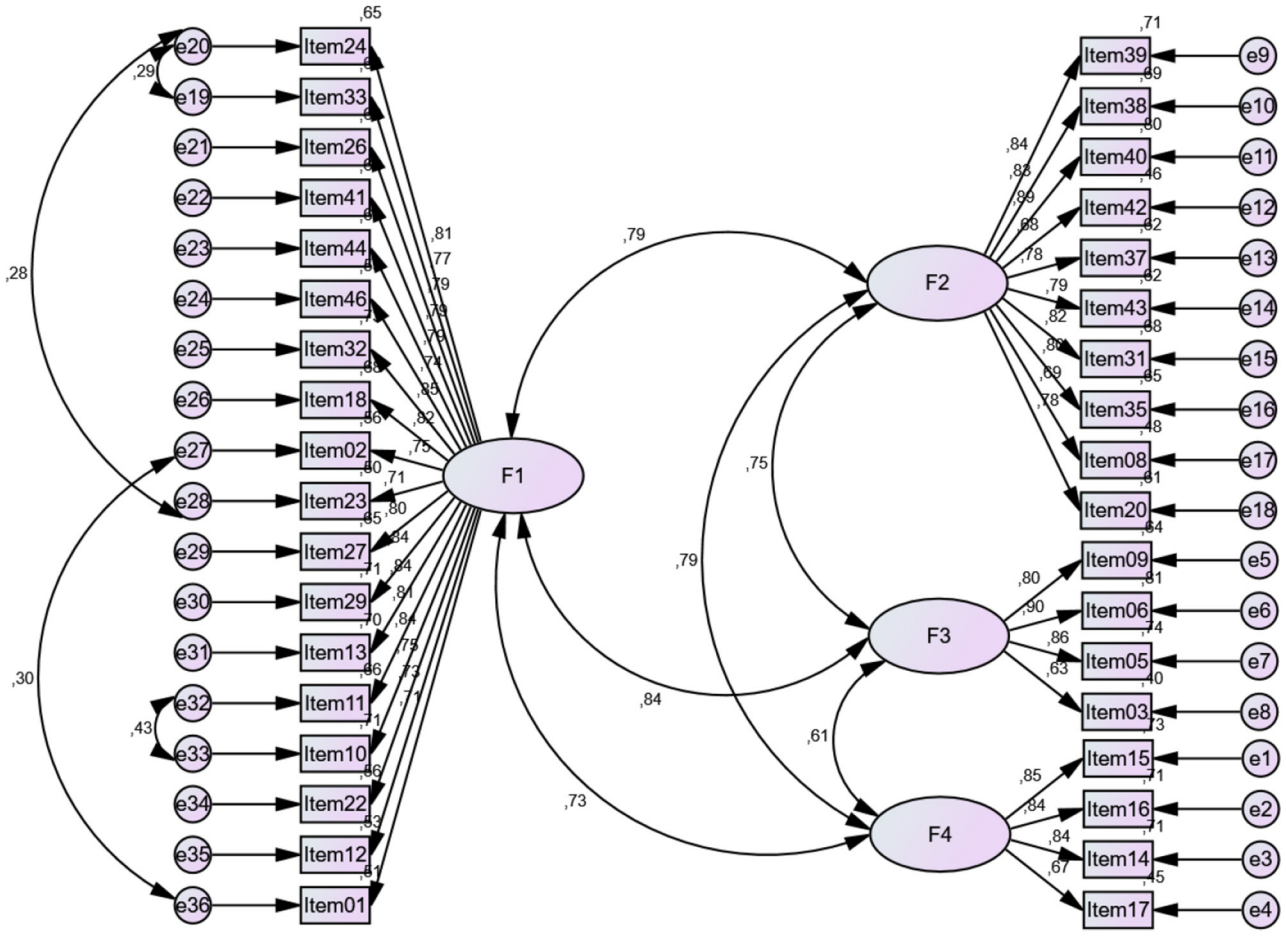
CFA output of measurement model.

The suitability of the four-factor structure that emerged as a result of the Exploratory Factor Analysis (EFA) was tested with Confirmatory Factor Analysis (CFA) with three models. Model 1 was first order four-factor uncorrelated model. Model 2 was first order four-factor correlated model and model 3 was first order four-factor correlated model with modifications. The fit indices retrieved as a result of testing all three models were reported in Table 3 and CFA output of measurement model 3 was illustrated in Figure 2.

**Table 3:** Goodness of fit indices and criterion values regarding the fitness of the measurement model of the TDC scale.

^**^Models	***X^2^***	***df***	***p***	***X^2^/df***	***RMSEA***	***CFI***	***IFI***	***NFI***	***Δ X^2^***	***Δ df***	***p***
^*^Acceptable fit				* < 5*	<0.08	>0.90	>0.90	>0.90			
^*^Very good fit				* < 3*	<0.05	>0.95	>0.95	>0.95			
*Model 1*	2,324.244	594	0.00	3.913	0.104	0.802	0.803	0.752			
*Model 2*	1,606.665	588	0.00	2.732	0.080	0.883	0.884	0.828	717,579	6	0.00
*Model 3*	1,480.693	584	0.00	2.535	0.075	0.897	0.898	0.842	843,551	10	0.00

^*^Sources: (Collier, 2020; Tabachnick and Fidell, 2019).

^**^Model 1: Four-Factor Model (First Order), Model 2: Four-Factor Correlated Model (First Order), Model 3: Four-Factor Correlated Model with modifications (First Order).

*X*^2^/*df* , RMSEA, NFI, CFI, IFI and CFI values were examined to determine the model fit of the alternative measurement model. The Δ *X*^2^ test were employed to understand if there is a significant change in the alternative goodness of fit indices for each model. The results of the model fit and criterion values are presented in Table 3.

A comparative analysis of model fit indices revealed that Model 1 exhibited the poorest fit [*X*^2^ = 2,324.244 *df* = 594, *p* = 0.00, *X*^2^/*df* = 3.913, RMSEA = 0.104, CFI = 0.802, IFI = 0.803, NFI = 0.752], with indices largely exceeding acceptable thresholds. In contrast, Model 3 outperformed the other two models producing indices [*X*^2^ = 1,480.693 *df* = 584, *p* = 0.00, *X*^2^/*df* = 2.535, RMSEA = 0.075, CFI = 0.897, IFI = 0.898, NFI = 0.842] that met or exceeded acceptability criteria. Based on the CFA results, the modified model fit values were at an acceptable level and the four-factor structure were supported and best fit with the dataset (Collier, 2020; Tabachnick and Fidell, 2019). Additionally, the Δ *X*^2^ test was performed to understand if there is a significant change in the alternative goodness of fit indices for each model. Results illustrated that both Model 2 (Δ *X*^2^ = 717,579) and Model 3 (Δ *X*^2^ = 843,551) were significantly performed better results compared with Model 1.

When the *t* and *R*^2^ values of the items belonging to the measurement model are examined in Table 4, the highest values were consecutively in the first factor, Item24 (*t* = 17.373, *R*^2^ = 0.806), in the second factor Item40 (*t* = 19.474, *R*^2^ = 0.892), in the third factor item Item6 (*t* = 17.135, *R*^2^ = 0.898), and in the fourth factor Item16 (*t* = 16.987, *R*^2^ = 0.844). These items make the highest contribution. These findings supported and validated the four-factor structure proposed in the EFA.

**Table 4:** Standardized *R*^2^ and *t* values for the items.

***F1***	***t***	***R^2^***	***F2***	***t***	***R^2^***	***F3***	***t***	***R^2^***	***F4***	***t***	***R^2^***
Item33	**-**	0.774	Item39	**-**	0.841	Item9	-	0.802	Item15	-	0.852
Item24	17,373	0.806	Item38	17,215	0.831	Item6	17.135	0.898	Item16	16.987	0.844
Item26	14,258	0.788	Item40	19,474	0.892	Item5	16.241	0.862	Item14	16.851	0.840
Item41	14,257	0.788	Item42	12,666	0.676	Item3	10.908	0.632	Item17	12.212	0.672
Item44	14,314	0.790	Item37	15,699	0.785						
Item46	13,276	0.744	Item43	15,832	0.789						
Item32	15,746	0.852	Item31	16,916	0.822						
Item18	15,093	0.824	Item35	16,318	0.804						
Item02	13,357	0.747	Item08	13,133	0.694						
Item23	12,469	0.706	Item20	15,559	0.780						
Item27	14,648	0.805									
Item29	15,585	0.845									
Item13	15,408	0.837									
Item11	14,792	0.811									
Item10	15,505	0.842									
Item22	13,436	0.751									
Item12	12,983	0.730									
Item01	12,619	0.713									

### 3.3 Reliability, discriminant and convergent validity analysis

To assess the reliability of the TDC scale, a comprehensive battery of psychometric measures was employed. While Cronbach's alpha coefficient is a commonly used method for assessing the reliability of multi-factor scales, its reliance on the assumption of unidimensionality has been widely criticized (Sijtsma, 2009). In recognition of this limitation, researchers have advocated for the use of multiple reliability indices to ensure the psychometric soundness of TDC scale. One such index is McDonald's omega (ω) coefficient. The ω coefficient is a more robust measure of reliability than Cronbach's alpha as it does not assume unidimensionality and is less affected by the number of items in a scale.

In addition to reliability, the establishment of discriminant validity is essential in research involving latent variables, particularly when multiple items or indicators are employed to operationalize constructs. This is to ensure that the latent constructs do not represent the same underlying phenomenon (Hamid et al., 2017). Discriminant validity is concerned with the extent to which a construct is distinct from other constructs. In this study, the composite reliability (CR) and average variance extracted (AVE) values were estimated to assess the convergent and discriminant validity of the latent structures of the TDC scale. CR, also known as internal consistency, is the combined reliability of the latent constructs that underlie the scale (Fornell and Larcker, 1981). The CR coefficient is a more robust measure of reliability than Cronbach's alpha as it takes into account both the factor loading values and error variances of the items (Sijtsma, 2009). Hair et al. (2019) suggested a CR value of 0.70 or higher as indicative of acceptable reliability. AVE is a measure of the amount of variance in the items of a construct that is explained by the construct itself. An AVE value of 0.50 or higher is generally considered indicative of acceptable convergent validity. The results of internal consistency, convergent and discriminant validity estimates and Heterotrait-Monotrait (HTMT) ratio of correlations between the constructs were presented in Table 5.

**Table 5:** Cronbach alpha (α), McDonald's omega (ω), CR, and HTMT ratios.

^*^Latent constructs	**α**	**ω**	CR^**^	AVE^**^	**HTMT correlation ratios**			
					**1**	**2**	**3**	**4**
(1) TPDC	0.966	0.967	0.966	0.620	1.0			
(2) TUICT	0.942	0.944	0.944	0.630	0.792^***^			
(3) TUHT	0.862	0.877	0.879	0.648	0.845^***^	0.776^***^		
(4) TDCD	0.874	0.878	0.880	0.649	0.801^***^	0.853^***^	0.762^***^	1.00

^*^TPDC, Teachers' Professional Digital Competence; TUICT, Teachers' Use of Instructional and Communication Tools; TUHT, Teachers' Use of Hardware Tools; TDCD, Teachers' Digital Content Development.

^**^CR and AVE values were estimated based on CFA results. ^***^p < 0.001.

As illustrated in Table 5, the Cronbach's alpha internal consistency coefficients calculated for the underlying constructs including TPDC (α = 0.97), TUICT (α = 0.94), TUHT (α = 0.86), and TDCD (α = 0.87), which indicated a good reliability regarding the internal consistency of the indicators (Hair et al., 1998). Additionally, McDonald's Omega (ω) coefficients, which are another internal consistency measure, are estimated values ranging from 0.97 to 0.88 across the constructs of TDC scale, which also supported the high-level internal consistency of the indicators. As shown in Table 5, all constructs exhibited CR values above the recommended threshold of 0.70 (Hair et al., 2019), ranging from 0.966 to 0.880. Additionally, all constructs exhibited AVE values above the recommended threshold of 0.50 (Fornell and Larcker, 1981), ranging from 0.649 to 0.620.

Convergent validity is a type of construct validity that assesses the extent to which a measure correlates with other measures of the same construct. In other words, it measures how well a measure captures the construct it is designed to assess. Convergent validity is demonstrated when a measure is highly correlated with other measures of the same construct, and when the items in the measure are all measuring the same thing (Hair, 2009). Two statistical indicators that can be used to assess convergent validity are CR and AVE. Hair (2009) suggests that convergent validity is observed when CR is higher than AVE, and AVE is higher than 0.5. In this study, all CR values are higher than estimated AVE values and all AVE values are higher than 0.5, which is an indication of the convergent validity of the latent constructs in the TDC scale. These findings suggest that the TDC scale constructs have good convergent validity, meaning that the items within each construct are well-correlated and measure the same underlying concept.

Discriminant validity was assessed using the HTMT ratio of correlations method (Henseler et al., 2015). The HTMT ratio is a modern approach to testing multicollinearity issues within latent constructs, and it is reportedly superior to Fornell and Larcker's (1981) method, which compares the square root of each AVE (Hamid et al., 2017). As proposed by Henseler et al. (2015), if the HTMT ratio is below 0.90, then discriminant validity has been established between two constructs. All HTMT ratios between the TDC scale constructs were below 0.90 while minimal concerns exist regarding the discriminant validity of the TUICT and TDCD constructs. The HTMT ratio between these two constructs were above 0.85. However, even this HTMT ratio was well below the threshold of 0.90, suggesting that discriminant validity can be considered acceptable for these two constructs in the measurement model. These results provide support for the distinctiveness of the TDC scale constructs (Henseler et al., 2015; Raykov, 1997). In conclusion, the TDC-S is a psychometrically sound measure of teachers' digital competences. This is evidenced by the strong convergent and discriminant validity of the constructs, as demonstrated by the CR, AVE, and HTMT ratio indices.

## 4 Discussion and conclusions

Digital competence is essential for teachers to effectively utilize digital educational tools, enhance the learning process, and engage students. It also contributes to teachers' professional development by enabling them to learn new teaching methods, collaborate with other teachers, and expand their professional networks (Basilotta-Gómez-Pablos et al., 2022; Garzón Artacho et al., 2020). In this regard, the present study aimed to develop a valid and reliable measurement tool to assess the digital competences of teachers. The sample group consisted of 271 teachers with a mean age of 40.61 ±7.57. As a result of the research, a five-point Likert-type scale consisting of a 36-item loaded onto four dimensions was developed, validated, and named as Teachers' Digital Competence Scale (TDC-S). The first factor consisting of 18 items was labeled as “Teachers' Professional Digital Competence (TPDC)”. The second factor, labeled as “Teachers' Use of Instructional and Communication Tools (TUICT)” consisted of 10 items, while the third factor, labeled as “Teachers' Use of Hardware Tools (TUHT)” consisted of four items. Finally, the fourth factor consisted of four items was labeled as “Teachers' Digital Content Development (TDCD)”. All these four factors mostly overlapped with the six dimensions of DigCompEdu. The total variance explained by four factors was 65.757%, which is beyond cut-off point for multi factor scales. Regarding the reliability analysis, internal consistency coefficients including Cronbach's Alpha, McDonald's Omega, CR and AVE were examined and all values validated the psychometric quality of the TDC-S. Confirmatory factor analysis (CFA) results showed that the four-factor structure proposed by exploratory factor analysis (EFA) was confirmed. All these results together confirmed the four-factor structure, resulting in TDC-S is a reliable and valid tool to assess teachers' digital competences.

Our research offers distinct theoretical and empirical contributions when juxtaposed with prior studies focused on the development of TDC scales (Alarcón et al., 2020; Al Khateeb, 2017; Cabero-Almenara et al., 2020a; Ghomi and Redecker, 2019; Gümüş and Kukul, 2023; Kuzminska et al., 2019; Tondeur et al., 2017). Among these, only three studies (Al Khateeb, 2017; Gümüş and Kukul, 2023; Tondeur et al., 2017) employed both EFA and CFA. In contrast, other studies either exclusively used CFA (Alarcón et al., 2020; Cabero-Almenara et al., 2020a; Ghomi and Redecker, 2019) or EFA (Kuzminska et al., 2019) to discern the primary components of the digital competence scale, such as Internet skills and Technology/ICT Literacy. Unlike many previous scale development studies, we utilized both EFA and CFA, and further enhanced its validity by assessing Composite Reliability (CR), Average Variance Extracted (AVE), and the Heterotrait-Monotrait (HTMT) correlation ratio. This rigorous approach arguably offers a more comprehensive and superior scale. As a result of the validity and reliability study of the TDC-S was rigorously conducted, it can be administered to all teachers at all levels and in different branches to measure their digital competences. Thus, TDC-S can be supportive in determining the digital competency level of teachers or prospective teachers. Additionally, our participant pool consisted of 271 teachers spanning a range of educational levels from primary to upper-secondary grades. This contrasts with previous studies, which either had limited sample sizes (Al Khateeb, 2017) or focused on teaching demographics, such as pre-service teachers (Tondeur et al., 2017), subject-specific teachers (Al Khateeb, 2017), or those in higher education (Cabero-Almenara et al., 2020a). Notably, although a limited number of studies (Alarcón et al., 2020; Ghomi and Redecker, 2019; Gümüş and Kukul, 2023) recruited a sample of teachers across all educational levels, their methodologies lacked the combined EFA and CFA approach, and/or the clarity of their convergent and discriminant validity measures for latent structures remains ambivalent. Lastly, our EFA results heralded that the total variance explained by all four factors in TDC-S surpassed that of other studies (Al Khateeb, 2017; Kuzminska et al., 2019; Tondeur et al., 2017), underscoring the robustness of TDC-S.

In addition to its theoretical and practical contributions to the field of TDC, our study has some limitations since the TDC-S tool was validated with a sample of teachers from a limited number of schools at different grade levels in Türkiye. Future research should therefore focus on adapting the TDC-S to other languages and countries, or on developing similar instruments in those countries, to enable cross-cultural comparisons and discussions. It would also be of interest to incorporate the views of students into instruments of this kind, regarding the digital competence of their educators and the digital resources available in their learning environment.

The development of the TDC-S measurement tool in this study is expected to add to the existing literature by evaluating TDC, which is essential for meeting individual needs. TDC-S will also serve as a valuable instrument for future research endeavors owing to its robust psychometric properties. The TDC-S holds the potential for broad applicability across diverse educational professionals and prospective teachers to assess their digital competencies effectively. This tool, validated through empirical research, offers a means to assess the digital proficiency of teachers and pre-service educators, enabling the identification of areas requiring further development. The TDC-S instrument stands poised to contribute to future studies aimed at evaluating and enhancing the digital competencies of educators, thereby supporting ongoing efforts to sustain teachers' continuous professional learning within the educational landscape. Furthermore, the validity and reliability of the scale can be tested with larger sample groups by applying it to different groups of teachers. Future research should also administer TDC-S and other existing digital competence assessment tools in order to test concurrent validity of the measures. In addition, TDC-S can be applied in future studies to evaluate multiple group differences in TDC-S scores according to gender, age, branch, and similar conditions. This could provide valuable insights into the factors that influence TDC. Such insights can be pivotal in reshaping teacher training curricula, ensuring the integration of digital competences into teaching-learning process in the digital era.

## Data availability statement

The raw data supporting the conclusions of this article will be made available by the authors, without undue reservation.

## Ethics statement

The studies involving humans were approved by Hitit University Ethical Board of Non-Interventional Studies (2021-88/10.01.2022). The studies were conducted in accordance with the local legislation and institutional requirements. The participants provided their written informed consent to participate in this study.

## Author contributions

MA: Writing – original draft, Writing – review & editing, Conceptualization, Data curation, Formal analysis, Investigation, Methodology, Project administration, Software, Supervision, Validation. TY: Data curation, Writing – original draft, Writing – review & editing. MK: Conceptualization, Validation, Writing – original draft, Writing – review & editing.
