# Supplementary material for: Teachers' digital competences: a scale construction and validation study
Source: Front Psychol. 2024 Aug 16;15:1356573. doi: 10.3389/fpsyg.2024.1356573 (PMC11363428; doi:10.3389/fpsyg.2024.1356573)
Supplement: Supplementary file 1 [file Table_1.docx]

# Appendix

The TDC-S final form.

|  | **Teachers’ Digital Competences Scale (TDC-S)**  Note: Here are some items to determine your level of digital competence. There is no correct answer, there is a suitable answer for you. Please take this into consideration and make your choice by putting an ( X ) sign in a single square in the relevant column without leaving any missing items. | **Strongly disagree** | **Disagree** | **Neither agree/ Nor disagree** | **Agree** | **Totally agree** |
| --- | --- | --- | --- | --- | --- | --- |
| **Items#** | **Factor 1: *“Teachers’ Professional Digital Competence”* (TPDC)** | **1** | **2** | **3** | **4** | **5** |
| Item 24 | I can create passwords and secure passcode for my personal accounts in the digital environment. |  |  |  |  |  |
| Item 33 | I can create secure passwords to protect my devices such as computers, smartphones and tablets. |  |  |  |  |  |
| Item 26 | I can read previously saved e-books, magazines, PDFs, articles in digital environment. |  |  |  |  |  |
| Item 41 | I can set up a digital group where I can communicate with students or parents. |  |  |  |  |  |
| Item 44 | I can use my e-mail account effectively (attaching files, creating an e-mail list, forwarding messages). |  |  |  |  |  |
| Item 46 | I can use learning management systems. |  |  |  |  |  |
| Item 32 | I can save files or content (e.g. text, pictures, music, videos, web pages) on the computer, tablet, and phone. |  |  |  |  |  |
| Item 18 | I can search an issue which I am curious about in online digital environment. |  |  |  |  |  |
| Item 02 | I can use digital devices with touch screens. |  |  |  |  |  |
| Item 23 | I know my rights and responsibilities regarding the confidentiality of personal data. |  |  |  |  |  |
| Item 27 | I can use CD/DVDs of publishers or books in offline digital environment. |  |  |  |  |  |
| Item 29 | I can use digital resources to make the classroom environment more interesting. |  |  |  |  |  |
| Item 13 | I can access the information which I needed in the digital environment by using various technological resources. |  |  |  |  |  |
| Item 11 | I can access internet resources in the digital environment via mobile devices. |  |  |  |  |  |
| Item 10 | I can use search engines effectively in digital environment. |  |  |  |  |  |
| Item 22 | I can exchange information with teachers in online environments (forums, video conferences, virtual social networks, etc.) to improve my teaching skills. |  |  |  |  |  |
| Item 12 | I can use educational web sites that support learning in digital environment. |  |  |  |  |  |
| Item 01 | I can use smart board in classroom environment. |  |  |  |  |  |
| **Items#** | **Factor 2: *“Teachers’ Use of Instructional and Communication Tools”* (TUICT)** | **1** | **2** | **3** | **4** | **5** |
| Item 39 | I can take notes on electronic books in digital environment. |  |  |  |  |  |
| Item 38 | I can convert documents into different formats. |  |  |  |  |  |
| Item 40 | I can use different presentation tools. |  |  |  |  |  |
| Item 42 | I can prepare a blog for educational purposes in digital environment. |  |  |  |  |  |
| Item 37 | I can add audio, video, graphics, and animations to my presentations. |  |  |  |  |  |
| Item 43 | I can use communication applications in digital environment. |  |  |  |  |  |
| Item 31 | I can produce materials needed for the course content in offline digital environments. |  |  |  |  |  |
| Item 35 | I can prepare video and audio files regarding the course content in digital environment. |  |  |  |  |  |
| Item 08 | I know the hardware features of the digital device which I bought or will buy. |  |  |  |  |  |
| Item 20 | I can compare information with many different resources to check for the reliability of information in online digital environment. |  |  |  |  |  |
| **Items#** | **Factor 3: *“Teachers’ Use of Hardware Tools”* (TUHT)** | **1** | **2** | **3** | **4** | **5** |
| Item 5 | I can use additional equipment (headphones, camera, printer, projection, external memory, etc.) by establishing a wired (USB, HDMI, VGA, etc.) connection with digital devices. |  |  |  |  |  |
| Item 6 | I can use additional equipment (headphones, camera, printer, projection, external memory, etc.) by establishing wireless (Wi-Fi, Bluetooth, etc.) connection with digital devices. |  |  |  |  |  |
| Item 9 | I can use various equipment related to digital technology (headphones, camera, printer, projection, external memory etc.) effectively. |  |  |  |  |  |
| Item 3 | I can use projector in classroom environment. |  |  |  |  |  |
| **Items#** | **Factor 4: *“Teachers’ Digital Content Development”* (TDCD)** | **1** | **2** | **3** | **4** | **5** |
| Item 15 | I can prepare material that will enable the student to reinforce the topic in digital environment. |  |  |  |  |  |
| Item 16 | I can produce materials that motivate students to learn in digital environment. |  |  |  |  |  |
| Item 14 | I can produce online materials required for course content in digital environments. |  |  |  |  |  |
| Item 17 | I can have my students play educational games in digital environment. |  |  |  |  |  |
